# Danlou Recipe promotes cholesterol efflux in macrophages RAW264.7 and reverses cholesterol transport in mice with hyperlipidemia induced by P407

**DOI:** 10.1186/s12906-023-04253-9

**Published:** 2023-12-08

**Authors:** Wenrun Han, Dandan Zhang, Peng Zhang, Qianqian Tao, Xiaoli Du, Chunquan Yu, Pengzhi Dong, Yan Zhu

**Affiliations:** 1 State Key Laboratory of Component-Based Chinese Medicine, Tianjin University of Traditional Chinese Medicine, 10 Poyanghu Road, Jinghai District, Tianjin, 301617 China; 2 Research and Development Center of Traditional Chinese Medicine, Tianjin International Joint Academy of Biomedicine, 220 Dongting Road, TEDA, Tianjin, 300457 China; 3 Department of Pharmacy, Inner Mongolia Medical College, Hohhot, 010110 China

**Keywords:** Ethanol extract of Danlou tablet, Liver X receptor, Reverse cholesterol transport, Hyperlipidemia, Traditional Chinese Medicine

## Abstract

**Introduction:**

Liver X Receptor (LXR) agonists could attenuate the development of atherosclerosis but bring excess lipid accumulation in the liver. Danlou Recipe was believed to be a benefit for improving the lipid profile. Thus, it is unclear whether Danlou Recipe could attenuate hyperlipidemia without excess lipid accumulated in the liver of mice. This study aimed to clarify if Danlou Recipe could alleviate the progression of hyperlipidemia in mice without extra lipids accumulated in the liver.

**Methods:**

Male murine macrophage RAW264.7 cells and murine peritoneal macrophages were used for the in vitro experiments. Cellular cholesterol efflux was determined using the fluorescent cholesterol labeling method. Those genes involved in lipid metabolism were evaluated by qRT‐PCR and western blotting respectively. In vivo, a mouse model of hyperlipidemia induced by P407 was used to figure out the effect of Danlou Recipe on reverse cholesterol transport (RCT) and hyperlipidemia. Ethanol extract of Danlou tablet (EEDL) was prepared by extracting the whole powder of Danlou Prescription from ethanol, and the chemical composition was analyzed by ultra-performance liquid chromatography (UPLC).

**Results:**

EEDL inhibits the formation of RAW264.7 macrophage-derived foam cells, and promotes ABCA1/apoA1 conducted cholesterol efflux in RAW264.7 macrophages and mouse peritoneal macrophages. In the P407-induced hyperlipidemia mouse model, oral administration of EEDL can promote RCT in vivo and improve fatty liver induced by a high-fat diet. Consistent with the findings in vitro, EEDL promotes RCT by upregulating the LXR activities.

**Conclusion:**

Our results demonstrate that EEDL has the potential for targeting RCT/LXR in the treatment of lipid metabolism disorders to be developed as a safe and effective therapy.

## Introduction

Hyperlipidemia, also referred to as dyslipidemia or high cholesterol, occurs when the metabolism of cholesterol is compromised, potentially resulting in various disorders. While cholesterol plays a crucial role in maintaining cell membrane fluidity and facilitating the production of hormones and bile acids, its disrupted metabolism can lead to adverse health conditions [1]. Generally, excessive lipid deposits will induce the transformation of macrophages to foam cells [2], which is considered to be the initiation of atherosclerosis [3]. Otherwise, lipid metabolism and lipotoxicity are the important inducers of liver diseases, such as non-alcoholic fatty liver disease (NAFLD) [4]. NAFLD encompasses a spectrum of liver conditions, starting from non-alcoholic fatty liver to nonalcoholic steatohepatitis (NASH), and in severe cases, it can progress to hepatic carcinoma. The primary pathological characteristics include the accumulation of excessive lipids in the liver and the presence of hepatic steatosis. However, NAFLD excludes conditions such as excessive alcohol consumption and other factors that could potentially trigger liver steatosis [5, 6]. Hyperlipidemia is thought to be a link between cardiovascular disease (CVD) and NAFLD. Pharmacological intervention targeting genes involved in cholesterol metabolism might be a benefit for the management of both diseases [7].

Reverse cholesterol transport (RCT) is a process that cholesterol is transported by high-density lipoprotein (HDL) from peripheral tissues back to the liver and ultimately excreted in bile and feces, which allows cells to export excess cholesterol and maintain homeostasis [8]. Lipid-activated nuclear receptors [9], LXRs, are master regulators of RCT and contribute to each RCT process. Recently, LXR has been thought to be associated with not only cholesterol metabolism but also inflammatory reactions and innate and adaptive immunity. This implies that LXRs might be a promising target for treating excess lipid accumulation in the aorta and liver [10, 11].

From a physiological standpoint, the activation of LXR plays a role in reducing the influx of macrophages into the arterial intima and preventing the uncontrolled accumulation of oxidized or modified cholesterol within foam cells, which are associated with atherosclerosis [12]. Meanwhile, LXRs promote cholesterol efflux from intimal macrophage foam cells. Although activated LXRs can regulate cholesterol metabolism and reduce inflammatory effects, LXRs agonists easily cause hepatic steatosis and dyslipidemia, which hampers the further development and application of synthetic LXRs agonists [13], such as LXRβ-selective agonist.

Numerous natural compounds are employed in the treatment of hyperlipidemia, targeting LXRs or LXR pathways to regulate lipid metabolism without promoting additional lipid accumulation. As a result, our research focuses on Traditional Chinese Medicine (TCM), which represents a vast resource of natural compounds, to elucidate its pharmacological effects in the management of hyperlipidemia [14]. Danlou prescription (DLP) originated from a classic Gualouxiebai Banxia Decoction, which was described in "The Synopsis of the Golden Chamber", an ancient Chinese medical book, and is currently widely used to cure cardiovascular diseases including coronary heart disease, atherosclerosis and hyperlipidemia [15]. DLP is composed of 10 herbs, *Trichosanthes kirilowii* Maxim.*, Allium macrostemon* Bunge*, Salvia miltiorrhiza* Bunge*, Astragalus membranaceus* Fisch. ex Bunge*, Ligusticum chuanxiong* Hort.*, Paeonia lactiflora* Pall.*, Curcuma aromatica* Salisb.*, Pueraria lobata* (Willd.) Ohwi*, Drynaria fortunei* (Kunze ex Mett.) J.Sm. and *Alisma orientale* (Sam.) Juz.. Studies have shown that DLP helps to eliminate phlegm, restore blood circulation, and improve hyperlipidemia [16]. Those abounded flavonoid derivatives in DLP, including puerarin, formononetin, and calycosin, have been reported to reduce cholesterol levels, and improve hypertension and thrombosis [17].

As the pharmacological mechanism of DLP is not yet fully clarified, this study was set to explore the therapeutic efficacy of ethanol extract of Danlou tablet (EEDL) for hyperlipidemia, as well as discuss the effect and mechanism of EEDL on RCT in vitro and in vivo.

## Materials and methods

### Drugs and reagents

Oxidized low-density lipoprotein (ox-LDL) (YB-002, Yiyuan Biotechnologies, China), T0901317 (T2320, MCE, China), Topflour cholesterol (810255P, Avanti Polar Lipids, USA), cholesterol (C3045, Sigma, USA), methyl-β-cyclodextrin (M102038, Aladdin, China), apoA1 (50918-M02H, Sino biological, China), 22-NBD-cholesterol (22-NBD-C, 30316, Cayman, USA), poloxamer P407 (P407, P2443, Sigma, USA), sodium oleate (S104196, Aladdin, China), hematoxylin (H3136, Sigma, USA), eosin (861006, Sigma, USA), and sodium palmitate (S161420, Aladdin, China) were purchased from commercial source and used in this study.

### Extract preparation

Danlou tablet, a patented drug, was purchased from the manufacturer (No. Z20040244, Cornell Pharmaceutical, Jilin, China). All the identification of Chinese medicinal materials as well as the classification of the plant of Danlou have been conducted by the manufacturer according to the Chinese Pharmacopoeia and taxonomic tables. EEDL was prepared according to the previous method and slightly improved [18]. Briefly, the whole Danlou tablet powder (100 g) was extracted with 70% ethanol (1 L) and ultrasonic water bathed for 30 min at room temperature (RT) and then filtered. This procedure was repeated three times and all the filtrate was pooled and concentrated into 100 ml on a rotary evaporator at 60°C. At last, the concentrate was lyophilized, and stored at four centigrade for use. Dulbecco’s modified eagle medium (12800017, DMEM, Gibco) was used to dissolve EEDL in this study. All EEDL used in vivo and in vitro was the same batch extraction from Danlou tablet.

### Qualitative chromatographic analysis

EEDL was analyzed on a Waters 2695 ultra-performance liquid chromatography (UPLC) system at detection wavelength of 280 nm (as a result, it was found that at a wavelength of 280 nm, a variety of exuding components can penetrate and provide effective components), using a Kromasil C18 Column (250 mm × 4.6 mm, five micronmeter, Sweden). The separation was performed at 40°C with a flow rate of one milliliter per minute. The mobile phase composition was water (0.1% formic acid, v/v, A) and acetonitrile (B). The protocol of elution was conducted as follows: 5–17% B at 0-7min, 17–25% B at 7–14 min, 25–28% B at 14–16 min, 28–30% B at 16–22 min, 30–85% B at 22–33 min, 85–95% B at 33-40min. The standard solution of ten chemicals (gallic acid, puerarin, daidzin, paeoniflorin, calycosin-7-O-glucoside, ferulic acid, naringin, salvianolic acid B, cryptotanshinone, and tanshinone IIA) was prepared in water or mobile phase, which was detected using the same UPLC method as EEDL for detection.

### Cell culture

RAW264.7 cells were obtained from ATCC (SC-6004) and cultured at 37℃ with 5% CO_2_ in DMEM medium added 1% P/S and 10% bovine calf serum. RAW264.7 cells were incubated with EEDL and ox-LDL at specified concentrations for 24 h, then the foam cell formation was identified by oil red O staining, which defines a foam cell by lipid droplets count per cell as greater than 10 [19]. HepG2 cells were purchased from ATCC (HB-8065) and cultured in RPMI 1640 medium (Gibco) with 10% FBS and 1% P/S. HepG2 cell was used as a cell-based model of NAFLD by oleic acid-palmitic acid (2:1), followed by EEDL treatment. The gene expression of lipid metabolism-related proteins in hepatocytes was detected by qualitative reverse transcription‐PCR (qRT‐PCR) or western blot 24 h after cell administration.

Wild type C57BL/6 mice (8-week males) were from National Institutes for Food and Drug Control (Beijing, China, Certificate no.: SCXK Jing, 2017–0005) and used to collect peritoneal macrophages. Briefly, mice were intraperitoneally injected with four ml 4% thioglycolate solution on day one. On day five, mice peritoneal macrophages were collected from the abdomen by lavage using cold PBS. Then, the obtained cells were incubated in complete DMEM medium for two hours. The adhesive macrophages were cultured in newly changed complete DMEM medium for an additional 48 h and were ready for further use [20].

### Establishment of animal model

All animal experiments in the study have been approved by the Animal Ethics Committee of Tianjin University of Traditional Chinese Medicine (TCM-LAEC2020096). The P407-induced mouse model of hyperlipidemia was conducted as described [21]. Briefly, the 8-week-old male C57BL/6 mice were allowed free access to a normal diet and water for one week, and then randomly assigned into five groups and 8 mice for each: control group, model group, EEDL-L group (100mg/kg body weight), EEDL-H group (400mg/kg body weight), T0901317 group (5 mg/kg). The control group was given a blank reagent by gavage, and the other groups were fed with high-fat food and injected with P407 (300 mg/kg body weight, twice a week) into the abdominal cavity. Gavage is administered according to the group, each other day, for 30 consecutive days.

### Oil red O staining

RAW264.7 cells were fixed (4% paraformaldehyde) for 20 min and then washed using PBS. The cells were infiltrated with 60% isopropanol for 10 s, and the oil red staining solution (oil red storage night (0.5%): high pressure ultrapure water = 3:2) dyeing at room temperature for one hour. Then, the adhesive cells were washed three times with PBS to remove background staining. Finally, microscope was used to observe the lipid formation of droplets in the cells. The foam cell formation was identified by oil red O staining, which defines a foam cell by lipid droplets count per cell as greater than 10 [19]. Meanwhile, isopropanol was used to extract lipids from the cells to quantify foam cell formation by recording the absorbance at 510 nm using a microplate reader (VICTOR® Nivo™, PerkinElmer, FIN).

### Immunofluorescence

RAW 264.7 cells were fixed (4% paraformaldehyde), permeabilized (0.1% Triton X-100) and blocked (5% BSA/PBS). To determine liver X receptor α (LXRα) protein expression, total protein extraction was incubated with anti-LXRα IgG (ab3585, rabbit polyclonal, 1:100) and goat-anti rabbit Alexa Fluor 647/488 (1:1000), subsequently. Besides, the nucleus was labeled with Hoechst 33,342 (1:300). High-content acquisition of immunofluorescence images and qualitative analysis were conducted using PerkinElmer Operetta system (USA).

### Cholesterol efflux test in vitro

RAW264.7 cells were plated in complete DMEM medium in 96-well plates at a density of 30000 cells/well for 16 h. Macrophages were labeled with Topflour cholesterol by incubating the monolayers with 0.2 ml of labeling medium containing methyl-β-cyclodextrin/Topflour cholesterol/unlabeled cholesterol for six hours, followed by washing with DMEM. RAW264.7 cells were then equilibrated with DMEM with 0.2% BSA for one hour. Then, the cells were washed with DMEM and incubated for 18 h with DMEM media containing apoA1 (10 µg/ml) and EEDL (100/200/400 µg/ml) or T0901317 (0.2/0.5 µM). The LXR agonist at indicated concentration in different assays. Next, the supernatant was removed, and the cell layer was added with lysate and shaken by a shaker at room temperature for 30 min. Finally, the fluorescence intensity of the cell lysate and supernatant was detected with a microplate reader (Ex/Em = 482 nm/516 nm) [22].

### Cholesterol efflux in vivo

To investigate the effect of EEDL on RCT in vivo, fluorescent cholesterol-loaded macrophages were used. Firstly, RAW264.7 were incubated with 22-NBD-C and then the incubated cells (22-NBD-C harbored) were intraperitoneally injected into P407-induced hyperlipidemia mice (4 × 10^6^ cell/mouse). All animals were fastened for 12 h before being sacrificed. Samples were collected 24 h after injection: blood was obtained from mouse eyes, the bile and the liver were dissected and rinsed in saline and then stored at -20℃ for use, and feces were gathered and lyophilized for subsequent studies. The fluorescent intensity of 22-NBD-C was recorded and calculated the percentage relative to the injection volume [23].

### Hematoxylin and eosin (H & E) staining

The mouse liver was fixed in 10% neutral formalin for 24 h followed by tissue processing and paraffin embedding. The paraffin block was prepared as four micrometer sections and stained with H&E. Histopathological analysis was carried out using an inverted fluorescence microscope (AZ100, Nikon, JPN).

### Enzyme-linked immunosorbent assay (ELISA)

The protein contents of cytochrome P450 family 7 subfamily. A member 1 (CYP7A1) in the mouse liver or serum were measured using Mouse CYP7A1 ELISA kit (ZC-38081, ZCIBIO, China) following the manufacturer’s instructions. The content of bile acid in the mouse liver or feces was measured using a bile acid kit (MAK309, Sigma, USA).

### Total RNA isolation and qRT-PCR assay

Trizol reagent was used to extract total RNA from cells or liver samples under the instructions of the manufacturer’s guidance (Invitrogen, Carlsbad, CA, USA). The NanoDrop™ Lite system (Thermo Fisher Scientific, Waltham, MA, USA) was used to determine the RNA concentration and purity. PrimeScript™ RT Reagent Kit was used to synthesis cDNA which was amplified later according to the protocols of GoTaq® qPCR Master Mix. The Light Cycler® 96 System (Roche Applied Science, Penzberg, Germany) was used to read the threshold cycle (Ct) and follow the re-action conditions: 95.0 ◦C for eight min, 45 cycles at 95.0 ^◦^C, 5 s, 60 ^◦^C, 30s. 2-^△△^CT method was used to calculate the fold changes of mRNA contents. Liver X receptor alpha (LXRα), liver X receptor beta (LXRβ)), sterol regulatory element-binding protein 1 (SREBP1c), cluster of differentiation 36 (CD36), scavenger receptor class B type 1 (SRB1), ATP binding cassette transporter A1 (ABCA1), ATP binding cassette G1 (ABCG1), ATP binding cassette G 5 (ABCG5), ATP binding cassette G8 (ABCG8), CYP7A1, low-density lipoprotein receptor (LDLR), inducible degrader of the low-density lipoprotein receptor (IDOL) and glyceraldehyde 3-phosphate dehydrogenase (GAPDH) primers for qRT-PCR were synthesized by Sangon Biotech (Shanghai, China) and the detailed information were listed in Table 1 (Human source) and Table 2 (Mouse source).

**Table 1:** Oligonucleotide primers for real-time RT-PCR (Human source)

Gene name	Primer	Sequence (5′–3′)	Product size (nt)
LXRα	Forward	CTTCAGAACCCACAGAGATCC	126
	Reverse	AGCTCAGAACATTGTAGTGGAA	126
SREBP1c	Forward	AGCAGCAGCAGCAGCAATGG	105
	Reverse	CGCCGAGGGAGAGAAGGAAGG	105
SRB1	Forward	GGAGATCCCTATCCCCTTCTAT	123
	Reverse	CTGAACTCCCTGTACACGTAG	123
ABCA1	Forward	TTTTTGCTCAGATTGTCTTGCC	126
	Reverse	TGTACTGTTCGTTGTACATCCA	126
ABCG1	Forward	CTCCTATGTCAGGTATGGGTTC	177
	Reverse	AAAATCCCGAGTACGATGAAGT	177
ABCG5	Forward	CAATGTGCTAAAGGGTGCTATC	101
	Reverse	GAAACAGATTCACAGCGTTCAG	101
ABCG8	Forward	GGTCACGGCGGCAAGATCAAG	135
	Reverse	GGTCTCTCGCACAGTCAAGTTGG	135
CYP7A1	Forward	GGAAAACCTCCAACGTATCATG	213
	Reverse	GGAAAGACTTTGTCGAATTGCT	213
LDLR	Forward	CTGTAGGGGTCTTTACGTGTTC	142
	Reverse	GTTTTCCTCGTCAGATTTGTCC	142
IDOL	Forward	ACATCAAAGGAGGTGTATGACC	82
	Reverse	TCTGGTTGTTTCTTGAAACGAG	82

**Table 2:** Oligonucleotide primers for real-time RT-PCR (Mouse source)

Gene name	Primer	Sequence (5′–3′)	Product Size (nt)
LXRα	Forward	GAGTGTCGACTTCGCAAATG	87
	Reverse	CTTCAGTTTCTTCAAGCGGATC	87
LXRβ	Forward	TCATCAATCCCATCTTCGAGTT	95
	Reverse	TGAGAAGATGTTGATGGCGATA	95
CD36	Forward	CTTTGAAAGAACTCTTGTGGGG	230
	Reverse	GTCTGTGCCATTAATCATGTCG	230
SRB1	Forward	AACATCACCTTCAATGACAACG	119
	Reverse	ACCAAGATGTTAGGCAGTACAA	119
ABCA1	Forward	TTTTTGCTCAGATTGTCTTGCC	126
	Reverse	TGTACTGTTCGTTGTACATCCA	126
ABCG1	Forward	CATGCTGCTGCCTCACCTCAC	87
	Reverse	TCTCGTCTGCCTTCATCCTTCTCC	87

### Western blotting

RIPA buffer (Sigma-Aldrich, St. Louis, MO, USA) with protease inhibitors cocktail was used to extract protein. All the homogenates were centrifuged at 12,000 g for 15 min and the supernatant was gathered. BCA protein concentration determination kit (PC0020, Solarbio, USA) was used to carry out the protein contents of cells or liver tissue. The PVDF membrane transferred with protein was then incubated with the antibodies LXRα (ab3585, rabbit polyclonal, 1:1000, Abcam, USA), LXRβ (60345–1-Ig, rabbit monoclonal, 1:2000, Proteintech, USA), CD36 (YT5585, rabbit polyclonal, 1:1000, Immunoway, USA) or SRB1 (NB400-104, rabbit polyclonal, 1:1000, Novus, USA) for 16 h at four centigrade. Then, the membrane was washed with TBST three times and incubated with horse-radish peroxidase-conjugated secondary anti-mouse or anti-rabbit IgG (1:3000, Immunoway, USA) at RT for two hours. ECL was used to develop the film.

### Statistical analysis

Experiments were carried out three times, independently. All values obtained in this study are represented as mean ± S.E.M. One-way ANOVA with Tukey correction was used to identify statistical significance. *P*-value < 0.05 was considered statistically significant.

## Results

### Chemical analysis of EEDL components

**Fig. 1:**
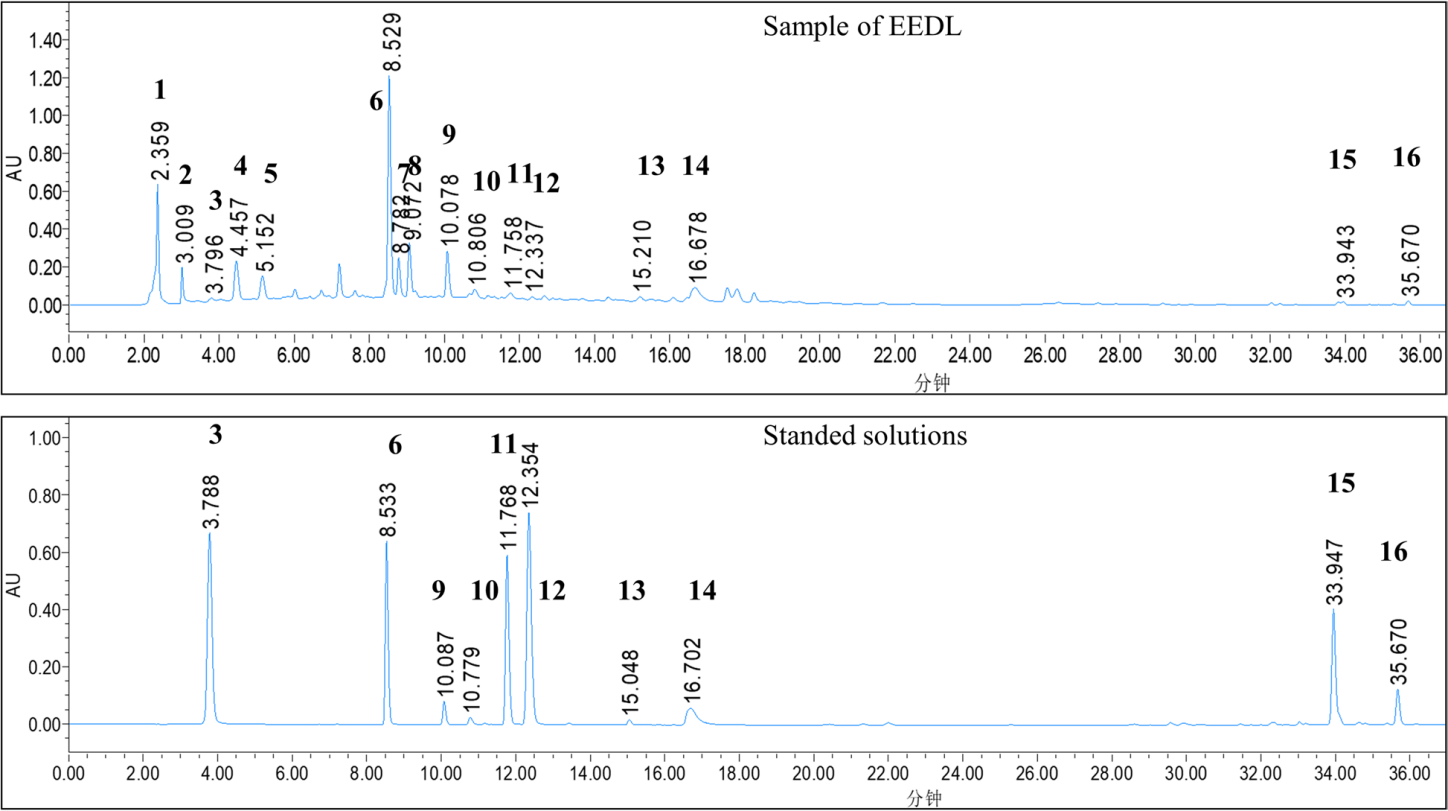
Ultra-performance liquid chromatography (UPLC) analysis of ethanol extract of Danlou tablet (EEDL). Standard mixture: “3” gallic acid; “6” puerarin; “9” daidzin; “10” paeoniflorin; “11” calycosin-7-O-glucoside; “12” ferulic acid; “13” naringin; “14” salvianolic acid B; “15” cryptotanshinone; and “16” tanshinone IIA

The UPLC analysis results of EEDL are shown in Fig. 1. By comparing retention time with the chromatogram of single standard chemicals, 10 peaks in standard solutions were determined, then ten compounds in EEDL were identified as gallic acid (3), puerarin (6), daidzin (9), paeoniflorin (10), calycosin-7-O-glucoside (11), ferulic acid (12), naringin (13), salvianolic acid B (14), cryptotanshinone (15), and tanshinone IIA (16).

### EEDL inhibits foam cell formation and promotes cholesterol efflux in vitro

**Fig. 2:**
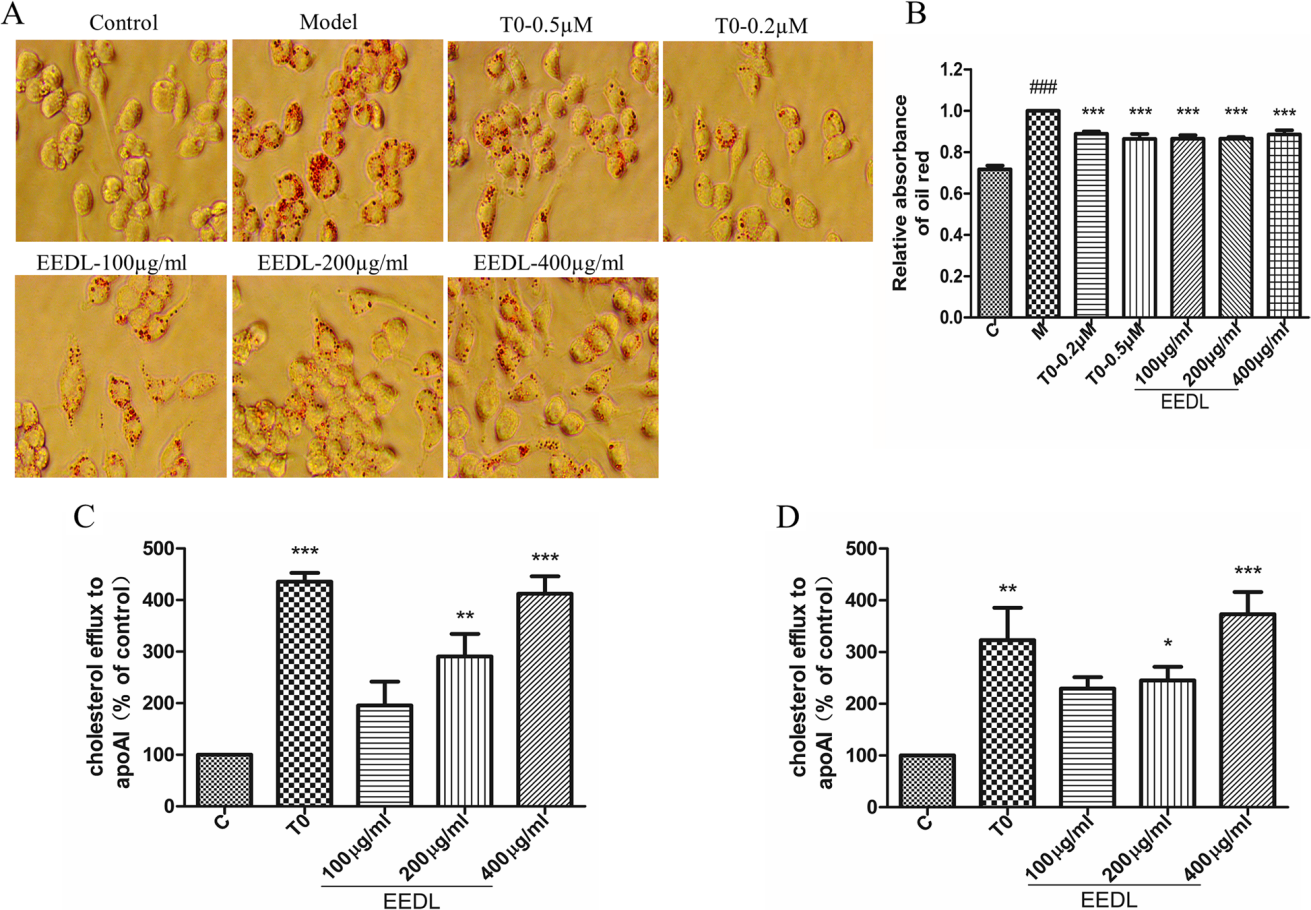
Effect of EEDL on the formation of macrophage foam cells and cholesterol efflux. **A** Representative images of oil red staining 24 h after drug administration. **B** Quantitation of oil red O staining. **C**-**D** Specific cholesterol efflux from RAW264.7 **C** and mouse peritoneal macrophages **D**. All the results are shown as the average value ± SD. **B**^###^*P* < 0.001 stands for experimental group vs. control group while ***P < 0.001 stands for experimental group vs. model group; **C**-**D** *P < 0.05, **P < 0.01, ***P < 0.001 stands for experimental group vs. Control group. T0 = T0901317

In order to evaluate the effect of EEDL on the formation of macrophage-derived foam cells, RAW264.7 cells were incubated with EEDL and ox-LDL at the specified concentration. Effects of EEDL on the formation of macrophage foam cells and cholesterol efflux were shown in Fig. 2. EEDL administration significantly reduced the intracellular lipid accumulation induced by ox-LDL (Fig. 2A and B). We next investigated the effect of EEDL on cholesterol efflux in RAW264.7 macrophages and mouse peritoneal macrophages. EEDL dose-dependently promotes the specific outflow of both intracellular cholesterol to apoA1 (Fig. 2C and D).

### EEDL promotes nuclear translocation of LXR in RAW264.7 macrophages and regulates LXR target proteins involved in lipid metabolism

**Fig. 3:**
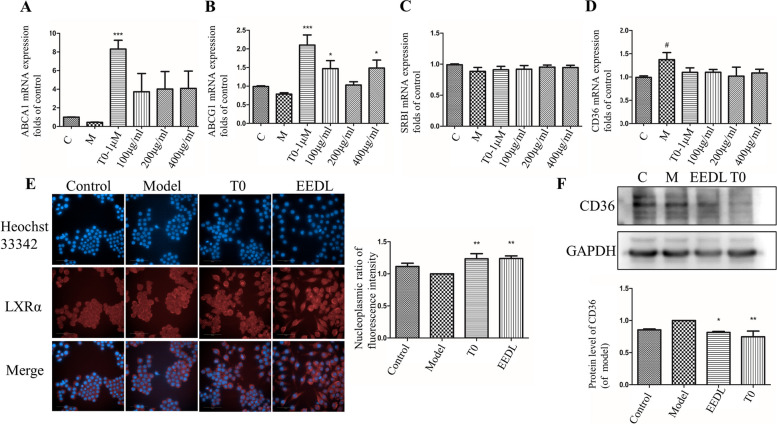
Effect of EEDL on liver X receptor (LXR) nuclear translocation and the mRNA levels of LXR regulated proteins in RAW264.7 macrophages. **A**-**D** The mRNA levels of ATP binding cassette subfamily A member 1 (ABCA1), ATP binding cassette subfamily G member 1 (ABCG1), scavenger receptor class B type 1 (SRB1) and transmembrane glycoprotein cluster of differentiation 36 (CD36). **E** Images of immunofluorescence 16 h after drug administration and the quantitation of fluorescence intensity. **F** The protein level of CD36. All the results are shown as average value ± SD. ^#^*P* < 0.05 stands for experimental group vs. control group while *P < 0.05, **P < 0.01, ***P < 0.001 stands for experimental group vs. model group. T0 = T0901317

LXRs regulates lipid homeostasis and plays an important role in regulation of reverse cholesterol transplant. Thus, we evaluated the effect of EEDL on LXR nuclear translocation and the mRNA levels of LXR regulated proteins in the ox-LDL-induced foam cell model. The effects of EEDL on LXR nuclear translocation in RAW 264.7 macrophages and the mRNA levels of LXR-regulated proteins were shown in Fig. 3. LXRs regulate lipid homeostasis and play an important role in regulation of reverse cholesterol transport. Thus, we evaluated the effect of EEDL on LXR nuclear translocation and the mRNA levels of LXR-regulated proteins in the ox-LDL-induced foam cell model. Compared to the model group, LXR translocation was significantly increased after EEDL administration (Fig. 3E). In addition, EEDL treatment increased gene expression of ABCA1 and ABCG1 while decreasing the mRNA and protein level of CD36 (Fig. 3A, B, D, F) in ox-LDL-stimulated RAW264.7 macrophages. Given the mRNA levels of SRB1, EEDL treatment had no significant effect (Fig. 3C).

### EEDL promotes reverse cholesterol transport in P-407-induced hyperlipidemia mice

**Fig. 4:**
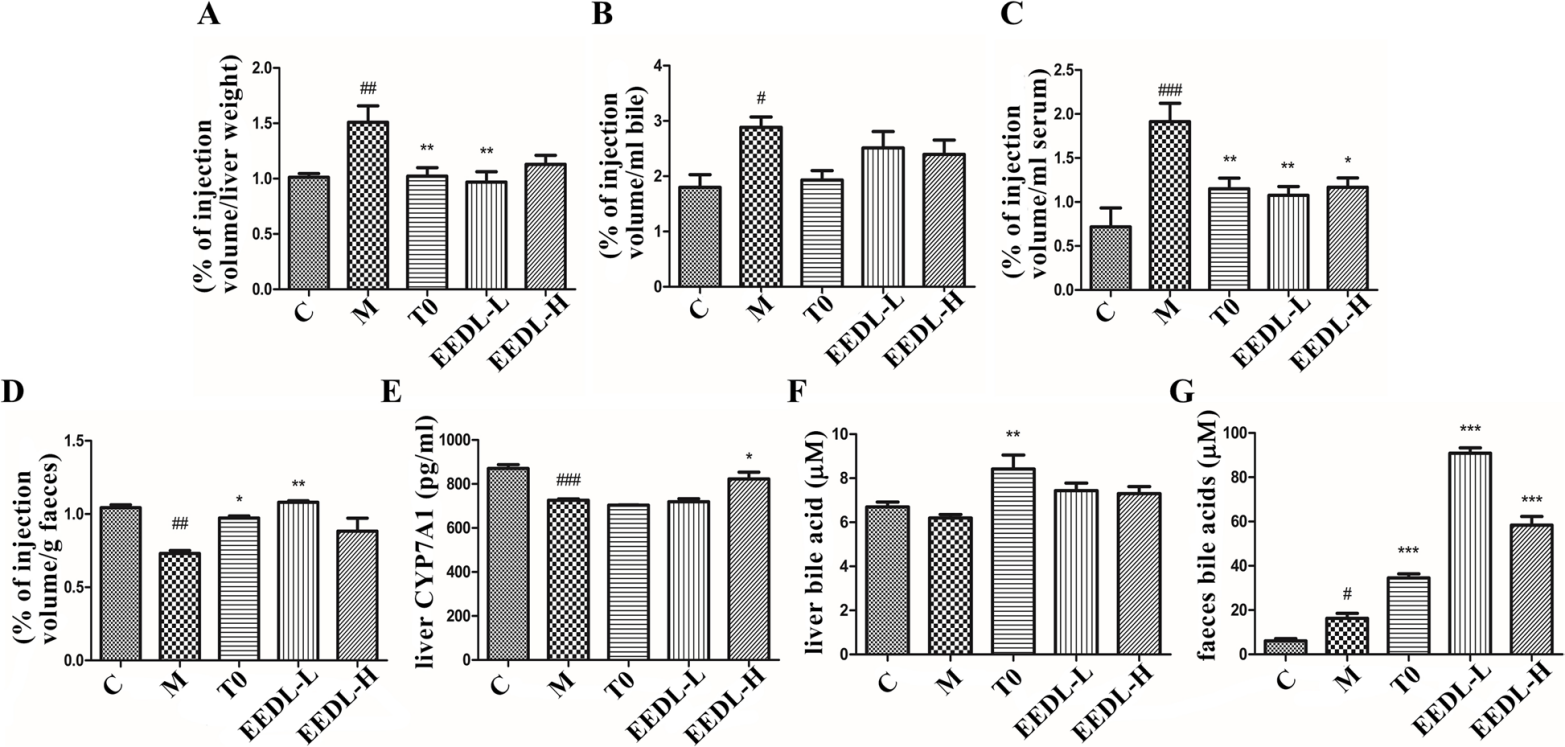
Effect of EEDL on reverse cholesterol transport (RCT) in P-407-induced hyperlipidemia mice. **A**-**D** The percentage of lipids extracted from liver, bile, serum, and feces as compared to the injection volume. **E** The level of cholesterol 7 alpha-hydroxylase (CYP7A1) in liver was determined by ELISA kit. **F**-**G** The amount of bile acids in the feces and liver. All the results are shown as average value ± SD. (*n* ≥ 3) ^#^*P* < 0.05, ^##^*P* < 0.05, ^###^*P* < 0.001 stands for experimental group vs. control group while *P < 0.05, **P < 0.01, ***P < 0.001 stands for experimental group vs. model group. T0 = T0901317

The effects of EEDL on reverse cholesterol transport in P-407-induced hyperlipidemia mice were shown in Fig. 4, which was examined using 22-NBD-C-loaded macrophages. Redistribution of the labeled cholesterol was detected 24 h after intraperitoneal injection of the above macrophages (Fig. 4). P407 notably promoted the labeled cholesterol gathering in the liver (Fig. 4A). Supplement of EEDL to P407-induced mice markedly decreased the 22-NBD-C levels both in mouse liver and serum (Fig. 4A, C) but no significant changes was found in the 22-NBD-C contents of bile among different groups (Fig. 4B). Meanwhile, P407 decreased the readouts of fecal fluorescence compared with the NC group (Fig. 4D). In addition, EEDL significantly increased the production of CYP7A1 in the liver and bile acid secretion in feces (Fig. 4E, G). Taken together, EEDL attenuated cholesterol accumulation in liver and plasma. Besides, EEDL increased fecal cholesterol secretion, thus upregulated macrophage-dependent RCT in vivo.

### EEDL improved liver damage and dyslipidemia in P407-induced hyperlipidemia mice

**Fig. 5:**
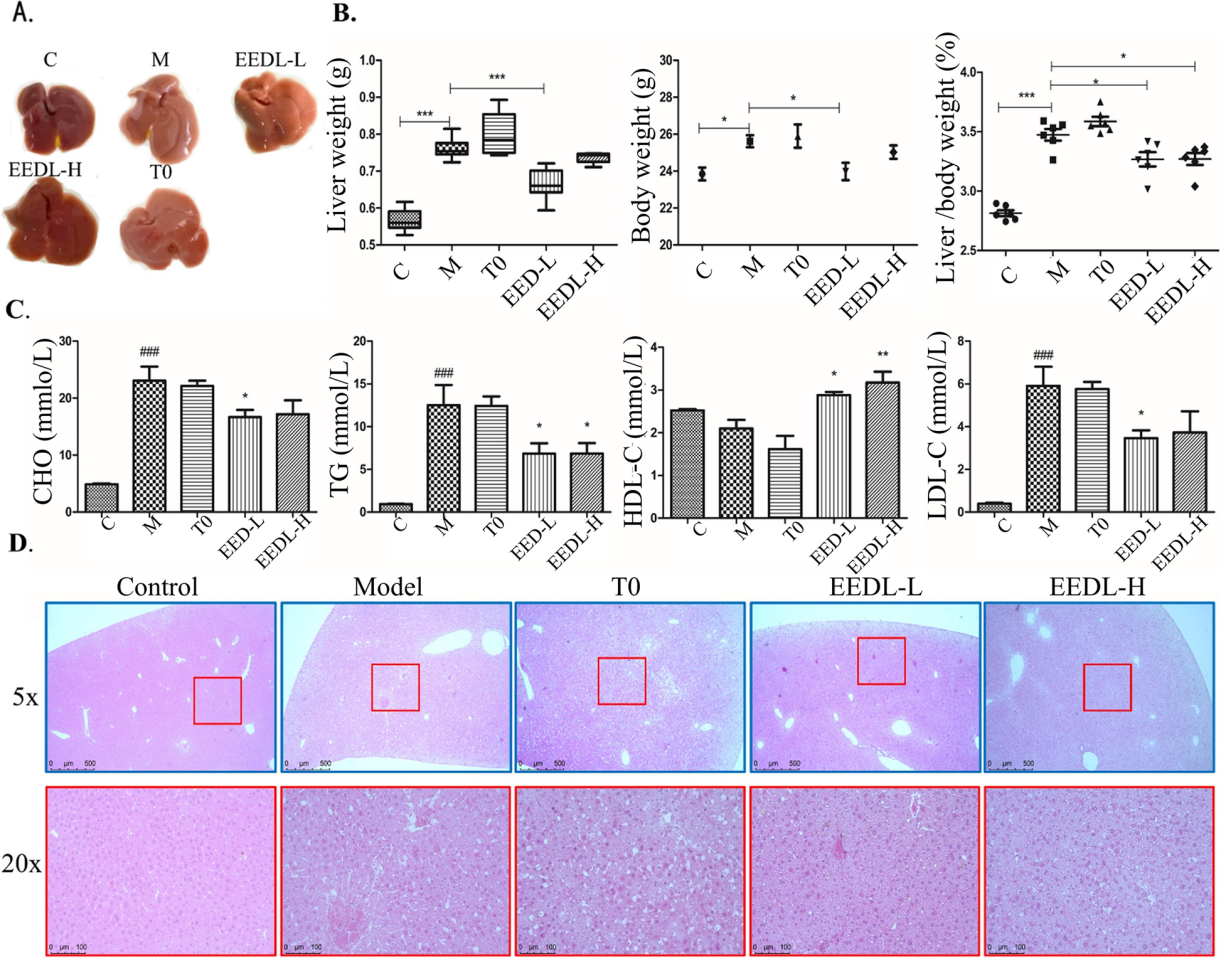
Effect of EEDL on liver damage and dyslipidemia in P-407-induced hyperlipidemia mice. The blood and liver samples were taken on day 30 after the drug administration and detected for various indicators. **A** Photographs of mouse liver from different treatment. **B** Liver weight, body weight, and ratio of liver to body weight of mice after 30 days of drug administration. **C** The levels of triglyceride (TG), total cholesterol (TC), High-density lipoprotein (HDL) cholesterol (HDL-C), LDL (low-density lipoprotein) cholesterol (LDL-C) in mouse serum. **D** Representative HE staining image of paraffin sections of mouse liver. All the results are shown as average value ± SD. (*n* ≥ 3) ^#^*P* < 0.05 or.^###^*P* < 0.001 stands for experimental group vs. control group while *P < 0.05, **P < 0.01, ***P < 0.001 stands for experimental group vs. model group. T0 = T0901317

P407 treatment induces the accumulation of triglycerides in mice which leads to hyperlipidemia. Effects of EEDL on liver damage and dyslipidemia were shown in Fig. 5. P407 treatment induces the accumulation of triglycerides in mice which leads to hyperlipidemia. After intraperitoneal injection of P407, the liver of C57 mice became whiter, larger in size, and increased in hepatosomatic ratio (Fig. 4A). However, the fatty liver of mice given EEDL by gavage significantly improved and hepatosomatic ratio decreased (Fig. 5A, B). In addition, P407 treatment resulted in a significant increase in serum triglyceride (TG), total cholesterol (TC), low-density lipoprotein cholesterol (LDL-C) contents while a decrease in HDL-C. After intragastric administration of EEDL, it significantly improved the dyslipidemia, liver damage and inflammatory infiltration induced by P407 (Fig. 5C, D).

### EEDL regulates the expression level of proteins related to hepatic lipid metabolism in vitro and in vivo.

**Fig. 6:**
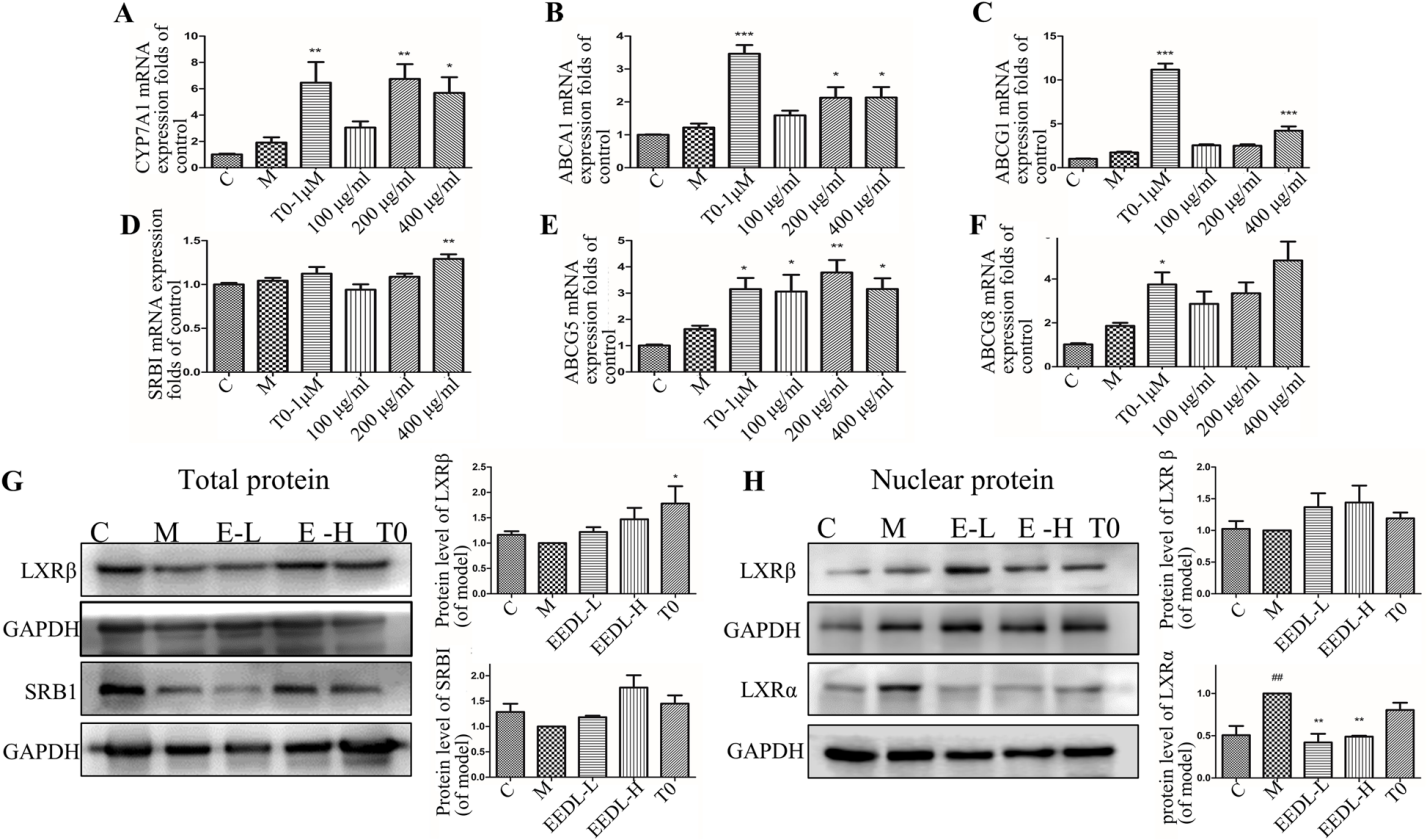
Effect of EEDL on the expression of proteins related to hepatic lipid metabolism in vitro and in vivo. **A**-**F** The mRNA levels of CYP7A1, ABCA1, ABCG1, SRB1, ATP binding cassette subfamily G Member 5 (ABCG5) and ATP binding cassette subfamily G Member 8 (ABCG8) in HepG2 cells. **G** The total protein levels of liver X receptor β (LXRβ), SRB1 and **H** The nuclear protein levels of LXRα/β in mouse liver. All the results are shown as average value ± SD. (*n* ≥ 3) ^##^*P* < 0.05 stands for experimental group vs. control group while *P < 0.05, **P < 0.01, ***P < 0.001 stands for experimental group vs. model group

The effect of EEDL on the expression of proteins related to hepatic lipid metabolism in vitro and in vivo is shown in Fig. 6. EEDL significantly increased the expression of CYP7A1 (Fig. 6A), which is a regulator of cholesterol metabolism. Meanwhile, EEDL significantly upregulated the expression of ABCA1, ABCG1, ABCG5 and ABCG8 (Fig. 6B, C, E) within HepG2 cells, thus promoting cholesterol transport while inhibiting cholesterol accumulation. As for cholesterol intake procedure, EEDL tended to increase SRB1 expression (Fig. 6D, G). As for LXRs, EEDL promoted the overall expression of LXRβ after fatty acid induction and increased the LXRβ protein contents in nuclear while reduces the contents of LXRα (Fig. 6G, H) in mouse liver.

## Discussion

Danlou Recipe was previously used in treating cardiovascular disease. In this study, we used P407 induced hyperlipidemia model and found EEDL could attenuate hyperlipidemia development via promoting RCT without extra lipid accumulation in liver.

Several LXRs-regulated genes, such as ABCA1, ABCG1, SR-BI, apoE and apoA1 are involved in the clearance of excess cholesterol in foam cells by HDL [24, 25]. ABCA1 and ABCG1 synergistically promote RCT: ABCA1 increases cholesterol efflux to apoA1 to form nascent HDL particles while ABCG1 mediates the efflux of intracellular cholesterol to exogenous HDL [26–28]. Cholesterol accumulation in ABCA1/G1 deficient myeloid cells activates the NLRP3 inflammasome promoting neutrophil infiltration and necrosis in atherosclerotic lesions [29]. Additionally, ABCA1 is the main pathway for cholesterol efflux in macrophages during RCT [26]. In the present study, EEDL decreased the formation of macrophage foam cells while increased cholesterol efflux to apoA1 and the mRNA levels of ABCA1 and ABCG1 in macrophages and hepatocytes, which indicated that EEDL inhibited the formation of foam cells by promoting cholesterol efflux, thus inhibited lipid accumulation [30].

LXRs regulate LDLR, IDOL, ABCA1 and ABCG1 within macrophages, inhibiting cholesterol uptake while promoting cholesterol efflux. Activated LXRs induce HDL formation, cholesterol efflux and biliary cholesterol excretion via ABCG5 and ABCG8 pathway in the liver [11, 31, 32] nonetheless limit intestine cholesterol absorption [33–35]. LXR agonists accelerated cholesterol converted into bile acids by CYP7A1 [33, 36, 37]. Next, HDL-associated cholesterol is cleared by hepatic SRB1 [38] or transferred to apoB100-containing lipoproteins by cholesteryl ester transfer protein (CETP) for TG exchange. Furthermore, activated LXRs suppresses cholesterol biosynthesis by inducing expression of genes including non-coding RNA LXR-induced sequence (Lexis) and E3 ubiquitin protein ligase RING finger protein 145 [35]. Based on these knowledge, assays testing the translocation of LXRs between the cytosol and nuclear part were often used to measure the activity of these receptors when receiving specific stimuli. In order to avoid manual intervention for the software recognizing nuclear area and the cell outline, we used default parameters including auto-focusing to capture the images of the cell conducted this calculation when using High-Content Screening system. If more LXR alpha translocated, there would be more light color gathered in the nuclear area.

Macrophages are known to bind modified LDL via CD36 receptors, which is linked to the release of inflammatory mediators, macrophage recruitment, and foam cell formation. To our knowledge, J774A.1 cell line was obtained from female mice while RAW264.7 cell line was from male mice. We considered that there might be gender differences between these two cell lines. As a matter of fact, we could not sure if this discrepancy would or not affect the RCT, so we used RAW264.7 cell line to conduct RCT assay. We had tried once bone marrow macrophages, the amount and the status of the macrophages varied often between the individuals. It might need more mice at 6–8 weeks to eliminate the influence of individual differences. For the convenience and the stability of the experimental result we used RAW cells.. In this study, EEDL accelerated LXR migration into the nucleus then decreased mRNA (Fig. 3D) and protein levels of CD36 (Fig. 3E) which were repeated at least three times independently suggesting that EEDL can inhibit the cholesterol uptake of cells. Then EEDL promotes RCT in P407-induced hyperlipidemia mice by inhibiting cholesterol accumulation in liver, serum and bile, and promoting cholesterol excretion through feces. Indeed, increased fluorescence signal should be observed in plasma, liver as well as feces at same session. It might need preliminary experiment to get more ideal outcome. In HepG2 cells, EEDL increased mRNA levels of ABCG5, ABCG8 and CYP7A1, thus it might be the reason that there were much more bile acid found in feces. In conclusion, EEDL could stimulate RCT to prevent excessive accumulation of cholesterol by LXR pathways. In terms of cholesterol, serum and liver contents decreased, while feces content increased. This might have been a result of not catching the right time window for fluorescence measurements. At some point in time, it is possible to observe increased signals in liver, serum, and feces, which might be earlier than when samples are collected. Moreover, we can see in Fig. 4E that the CYP7A1 enzyme content had increased in the EEDL-H group. However, the bile acid content had increased in the liver as well (Fig. 4F) although not significantly.

Injection of P407 into mice produces severe hypertriglyceridemia (HTG) because it directly inhibits lipoprotein lipase (LPL)’s enzyme activity can be used to investigate the effects of HTG on atherosclerosis and the potential role of monocytes in these processes [39]. As a result of P407 injections, circulating monocytes accumulated lipids more rapidly. In this study, we used this model to mimic hyperlipidemia symptoms and expect whether EEDL could improve RCT in simulated disease status compared with wild type mice fed with high-fat diet. As a matter of fact, it had been well documented that P407 injection could induce hyperlipidemia [40]. In this study, lipid content in liver had been improved (Fig. 5) which was considered as an extra benefit when using EEDL to treat hyperlipidemia. This led us focused more on liver lipid metabolism other than hyperlipidemia and atherosclerosis development. Thus, a disease model of hyperlipidemia had been established and we considered it might be more appropriate than wild type to examine the EEDL pharmacological effect. To be more rigorous, RCT should be conducted in wild type mice as a baseline reference.

LXRs play important roles in hepatic lipogenesis. LXR agonists induces key genes (including SREBP1c, FAS, and ACC, etc.) which are critical for de novo lipogenesis [41]. Besides, activated LXRs also increases fatty acid biosynthesis by upregulating either the expression or activation of SREBP1c, stearoyl-coenzyme A desaturase 1 (SCD1), and fatty acid synthase. As reported, activation of LXRs induce both carbohydrate response element-binding protein and SREBP1c expression [42]. Whether EEDL could affect SREBPs expression would be an interesting issue for identifying EEDL pharmacological effect on regulating LXRs, based on the observation that “normal” colored liver gained than “white liver” treated by T0901317. In our study, EEDL showed some pharmacological similarity with LXR alpha agonist, T0901317. However, they do not behave all the same. Actually, we also tested the lipid content in HepG2 and HepRG cell line, there were no significant difference between model group and EEDLs treating group while T0901317 significantly increased the lipid accumulation (data not shown) which align with the finding in Fig. 5A. This might show that hyperlipidemia mice gained extra benefit from suffering fatty liver. Besides, activated LXRs can increase very low density lipoprotein particle secretion [43]. Activation of hepatic LXRs, however, hinders the development of LXR agonists due to unexpected activation of SREBP-1c. Thus, selectively activation of LXR has become an effective way to attenuate the development of atherosclerosis. As LXRα is predominantly found in liver, a reasonable method is to develop agonist selectively activate LXRβ [44]. Interestingly, EEDL was found to increase nuclear contents of LXRβ protein while decrease that of LXRα in vivo (Fig. 6H). We suspected LXR beta might play more important role in liver tissue than LXR alpha does when mice were feed EEDL. Although LXR alpha and LXR beta shared sequence, the two genes displayed differential tissue specificity as well as molecular function.

While steatosis is not considered harmful, its transition to NASH is considered a sign of further liver damage [45]. LXRs are believed to have a dual action in NAFLD. Activated LXRs can inhibit inflammatory actions within the liver and improve hypercholesterolemia [46–48]. The Kupffer cells and the hepatic stellate cells are thought to be the pivotal player for NASH, but LXRs activated within Kupffer cells/macrophages can inhibit acute hepatic inflammatory reactions [49]. LXRs inverse agonists decrease de novo lipogenesis and thus ameliorate lipotoxic injury, which in turn alleviate fibrosis and inflammatory reactions [10]. In a recent study, researchers discovered that SR9243, a liver-specific inverse agonist of LXRs, effectively alleviated fibrosis and inflammation in the liver. Additionally, it was found to reduce serum glucose levels and lower plasma lipid levels in a mouse model of high-cholesterol-induced NASH [50]. In this study, P407-induced fatty liver in mice along with inflammatory cell infiltrated and other liver damage. After EEDL administration, the hepatosomatic ratio of mice was significantly decreased and the dyslipidemia and inflammatory cell infiltration were also improved which suggested a protective role of EEDL in this model.

Those compounds identified in EEDL have been reported might contribute to attenuate the development of hyperlipidemia, atherosclerosis and NAFLD. The anti-hyperlipidemic action of gallic acid in *Emblica officinalis* was mediated by upregulation of PPARs, Glut4 and lipogenic enzymes, and decreased expression of proprotein convertase subtilisin/kexin type 9 (PCSK9) [51]. Ferulic acid ameliorates NAFLD by inhibiting free fatty acid uptake via the histone deacetylase 1 (HDAC1) / peroxisome proliferator- activated receptor gamma (PPAR-γ or PPARG) axis, which may provide potential dietary sdupplements and drugs [52]. Besides, the peroxisome PPARs, carnitine palmitoyltransferase 1A (CPT1A), acyl-coA oxidase 1 (ACOX1), and 3-hydroxy-3-methylglutaryl-coA synthase 2 (HMGCS2) are increased by ferulic acid treatment [53]. As for salvianolic acid B, it was reported that apoptosis and mitophagy are downregulated by salvianolic acid B in diabetic endothelial and mitochondrial cells [54]. By inhibiting chemically and mechanically activated Piezo1 channels, salvianolic acid B ameliorates atherosclerosis [55]. In ob/ob mice, salvianolic acid B also could inhibit hepatic lipid accumulation and NLR family pyrin domain containing 3(NLRP3) inflammasome [56]. In lipopolysaccharide/cigarette smoke-induced mice, naringin suppressed airway inflammation and improved pulmonary endothelial hyperpermeability [57]. In apoE^−/−^ mice, naringin inhibits cholesterol metabolism involved in gut microbiota remodeling [58]. Puerarin, also identified in *Pueraria lobata*, inhibits vascular smooth muscle proliferation and inflammation in atherosclerosis via lowering the expression of α-SMA and the inflammatory proteins IL-6 and IL-8. [59]. Besides, by regulating the AMPK pathway, puerarin improves hepatic glucose and lipid homeostasis [60]. However, in macrophages, puerarin modulates protein kinase RNA-like ER kinase (PERK) PERK/ the nuclear factor erythroid 2–related factor 2 (Nrf2) that coordinates with activating transcription factor 4 (ATF4) to activate thioredoxin 1 (Trx1), which reduces SR-A and lox-1 leading to inhibited lipid uptake [61]. This result reminds us it necessary to consider the multiple effects of EEDL improving hyperlipidemia when examining the specific pharmacological effect of the identified compounds. Daidzein promotes functional recovery in chronic stroke by enhancing cholesterol homeostasis via apoE gene [62]. Taken together, all the compounds found in EEDL were more or less associated with inhibited inflammatory activity, improved lipid metabolism. However, the relative content of each compound in EEDL should be considered that there might have combined effects using the whole recipe compared to single compound usage.

## Conclusion

In summary, EEDL promoted RCT and alleviated the disease progression of hyperlipidemia mice via mediating the LXRs pathway. And especially, EEDL selectively activate LXRβ in the liver which was further shown that it has protective effect on fatty liver induced by hyperlipidemia. These results indicated that EEDL might have the potential as an agonist of LXR to provide experimental evidence for the development of drugs targeting RCT or LXRβ.

## Data Availability

The datasets generated and/or analysed during the current study are included in the manuscript and raw data are available in the Baidu Netdisk repository. Please copy the link in the search bar of a browser and input the code below by following the prompts. Link: https://pan.baidu.com/s/1cHiPAQS8cMCnwJlwT2PfNw Code: 0ite.

## Abbreviations

22-NBD-C: 22-NBD-cholesterol
apoA1: Apolipoprotein A1
ABCA1: ATP binding cassette transporter A1
ABCG1: ATP binding cassette transporter G1
ABCG5: ATP binding cassette transporter G5
ABCG8: ATP binding cassette transporter G8
CD36: Cluster of differentiation
CYP7A1: Cytochrome P450 family 7 subfamily A 3member 1
EEDL: Ethanol extract of Danlou tablet
HDL: High-density lipoprotein
H&E: Hematoxylin and eosin
IDOL: Inducible degrader of the low-density lipoprotein receptor
LXR: Liver X receptor
LXRα: Liver X receptor α
LXRβ: Liver X receptor β
LCFA: Long-chain fatty acid
LDL: Low-density lipoprotein
LDL-C: Low-density lipoprotein cholesterol
LDLR: Low-density lipoprotein receptor
P407: Poloxamer P407
OA: Oleic acid
PA: Palmitic acid
RCT: Reverse cholesterol transport
RT: Room temperature
SRB1: Scavenger Receptor B1
SREBP1c: Sterol regulatory element-binding protein 1
TC: Total cholesterol
TCM: Traditional Chinese medicine
TG: Triglyceride
UPLC: Ultra-performance liquid chromatography
VLDL: Very-low-density lipoprotein
